# Socio-Economic Status: A Barrier to Access to Mandibular Advancement Device Therapy for Patients with Obstructive Sleep Apnea Syndrome in France

**DOI:** 10.1371/journal.pone.0138689

**Published:** 2015-09-24

**Authors:** Marion Fleury, Marc Le Vaillant, Nathalie Pelletier-Fleury, IRSR sleep cohort group

**Affiliations:** 1 Centre de Recherche Médecine Sciences, Santé, Santé mentale, Société, UMR 8211 (CNRS - INSERM - EHESS - Université Paris Descartes), Paris, France; 2 Equipe 1 'Economie de la santé - Recherche sur les services de santé', Centre de Recherche en Epidémiologie et Santé des Populations, UMR 1018 (INSERM - Université Paris Saclay), Paris, France; Oasi Institute for Research and Prevention of Mental Retardation, ITALY

## Abstract

**Background:**

Obstructive sleep apnea syndrome (OSAS) is a major public health problem which affects between 5 to 10% of the general population. OSAS is known to be associated with high rates of morbidity and mortality mainly due to cardiovascular diseases and traffic accidents. The burden of illness is high for the individual and society. There are 2 treatment options for OSAS, Continuous Positive Airway Pressure (CPAP) and Mandibular Advancement Device therapy (MAD). CPAP is known to be an effective but very constraining treatment. Patients are usually poorly adherent. MAD is a more recent treatment easier to use and consequently better tolerated, but MAD can only be prescribed to patients with satisfactory oral hygiene. Oral health constitutes a real issue particularly among underprivileged groups in France. Through this link, the question of whether low socio-economic status constitutes a barrier to access to care for patients with OSAS is raised.

**Methods and Principal Findings:**

In a multicenter prospective cohort of 2822 consecutive OSAS patients in whom MAD has been proposed as an alternative to CPAP between May 15, 2007 and December 1st, 2014, we identified the factors that lead to a patient diagnosed with OSAS to be treated by MAD instead of CPAP. A logistic regression was performed using a stepwise forward procedure. The main outcome of the study was that treatment by MAD was significantly associated with both educational attainment, as determined by the age at which the patient left full-time education, ≥18 years compared with <18 (adjusted odds ratio (aOR): 1.64, 95% CI 1.23 to 2.20), and the patient's occupational category. Executives and higher intellectual professions, intermediate professions, technicians, foremen and employees were significantly more likely to be treated by MAD than workers (aOR: 2.21, 95% CI 1.88 to 2.58; aOR: 1.74, 95% CI 1.15 to 2.63; aOR: 1.96, 95% CI 1.11 to 3.47, respectively).

**Conclusions:**

Overall, these results suggest that low socio-economic status constitutes a barrier to access to MAD for patients with OSAS in France. MAD use in patients with OSAS highlights inequalities in health care access.

## Introduction

Obstructive sleep apnea-hypopnea syndrome (OSAHS) is a common disease characterized by recurrent episodes of partial (hypopneas) or complete (apneas) collapse of the upper airway during sleep. Based on recent data from the Wisconsin sleep cohort study, it is estimated that currently among adults 30–70 years of age, approximately 14% of men and 5% of women have an apnea-hypopnea index (AHI) ≥ 5 plus symptoms of daytime sleepiness [1]. In France, the prevalence of symptoms evocative of obstructive sleep apnea is estimated at 4.9% (95% CI: 4.5–5.3) in the French general population aged over 18 years. [2]. The apnea/hypopnea index (AHI), ie, the number of apneas and hypopneas per hour of sleep, reflects the degree of severity of the disease. AHI of 5 to 15, 15 to 30 and more than 30 correspond to a mild, moderate and severe OSAHS, respectively. OSAHS can have a short-term and long-term impact on the patient depending on the severity of the disease. In the short term, sleep is not restorative and the subject experiences daytime sleepiness. In the long term, daytime sleepiness causes persistent fatigue, impaired alertness and memory disorders. Patients diagnosed with OSAHS have a 1.2- to 4.9-fold higher risk of motor vehicle accidents than disease-free individuals [3]. OSAHS is also responsible for cardiac arrhythmia and high blood pressure with possible significant long-term complications such as coronary heart disease (angina pectoris, myocardial infarction) and stroke [4]. Untreated OSAHS significantly increases the risk of fatal (odds ratio 2.87, 95%CI 1.17–7.51) and non-fatal (3.17, 1.12–7.51) cardiovascular events compared with healthy participants [5].

Continuous Positive Airway Pressure (CPAP) is the reference treatment for OSAHS [6] and has an undeniable effectiveness [7], particularly for patients with severe OSAHS. In contrast, the results of CPAP are less convincing in subjects with an AHI less than 30 [8]. CPAP consists of a turbine that propels ambient air under pressure into the respiratory tract via a tube attached to a mask placed over the individual’s nose. The positive air pressure eliminates apneas, and hypopneas as well, and prevents upper airway collapse and narrowing, reducing airflow limitations. This treatment can only reduce daytime sleepiness and associated comorbidities when the patient uses CPAP for more than 4 hours per night. However, CPAP is not universally accepted by users [9]. Only 46% to 80% of the treated population uses CPAP regularly (for more than 4 consecutive hours on 2 out of 3 nights) [10].

Oral appliance (MAD) therapy is a more recent treatment that can replace CPAP under certain conditions. It consists of a dental appliance generally composed of two parts: one fitted on the upper jaw and the other fitted on the lower jaw. The two parts of the appliance are connected to each other by a small lever to advance the mandible forward, opening the oral pharynx space, and allowing better air flow through the airways. Mandibular advancement can be adjusted according to patient tolerance to prevent apnea. This device is designed, prepared and fitted in the patient’s mouth by a dentist. A review of the literature of the effectiveness of this treatment has been published and demonstrated that the MAD can also reduce comorbidities and all other health consequences of OSAHS in the majority of non-obese patients [11]. This device is easier to use and patients’ adherence is usually high [12]. MAD can be proposed as first-line treatment in patients with moderate AHI, or regardless of the degree of severity when the patient refuses or does not tolerate CPAP [13].

However, although the cost of the device is reimbursed by national health insurance, MAD is not prescribed as often as it could or should be. In a recent paper, several barriers were identified which appear to limit the use of MAD [14]. These barriers are related to organizational issues, industrial development, contraindications (CIs) and side effects. CIs require particular attention, as the mechanical principle of the MAD is to maintain forced propulsion of the mandible in order to clear the airway during sleep. Stretching of the muscles and ligaments around the mandible induces certain constrains that are transmitted through the MAD to the teeth and temporomandibular joints (TMJ). When a mandibular advancement device is envisaged as treatment for OSAHS, the following CIs should be taken into account: (1) a sufficient number of teeth in each arch is required (n = 8), particularly posterior teeth to obtain solid intraoral fixation of the device; (2) untreated periodontal disease or substantial tooth mobility are CIs due to the risk of dental mobilization; (3) TMJ conflict is considered to be an absolute contraindication, as the device displaces the mandible anteriorly, inducing a constraint on the TMJ during sleep [15].

Part of the explanation for underuse of MAD is that oral health constitutes a real problem, particularly among underprivileged groups in France, as in other industrialized countries [16]. After the age of 50 years, the average number of missing, unreplaced teeth was 2.7 in men in the general population, versus 11.7 for men consulting health care centers for those in need [17]. This difference can be explained by different behavioral factors (dental hygiene, type of nutrition, etc.) [16], and also by inequalities in access to care [18]. Inequalities in access to care, more specifically access to dental care, are mainly due to low income and poor insurance coverage [19]. In several countries, healthcare systems do not cover or only partially (e.g. France) cover dental care. Forgoing dental care due to financial hardship has been shown to be associated with poorer oral health in several previous reports [20]. In the report published in France, workers were actually 1.8 times more likely to renounce dental care compared to executives, and workers without private health insurance were 2 times more likely to renounce dental care compared to those with private health insurance [19].

Little is known about the determinants of MAD treatment in patients with OSAHS. Are the indications guided exclusively by clinical criteria? Do socioeconomic factors play a role? The present study was based on the hypothesis that access to MAD therapy could be limited by poor socioeconomic conditions. The aim of the article is to identify the factors that lead to a patient diagnosed with OSAHS to be treated by MAD instead of CPAP.

## Materials and Methods

### Ethics statement

This multicenter cross-sectional study was conducted on the “Institut de Recherche en Santé Respiratoire des Pays de la Loire” (IRSR) sleep cohort. Approval was obtained from the University of Angers ethics committee and the “Comité Consultatif sur le Traitement de l’Information en matière de Recherche dans le domaine de la Santé” (C.C.T.I.R.S.) (07.207 bis). The database is anonymous and complies with the restrictive requirements of the “Commission Nationale Informatique et Liberté” (C.N.I.L.), the French personal data protection authority. All patients included in the IRSR sleep cohort provided their written informed consent.

### Design and study population

Since May 15, 2007, consecutive patients over the age of 18 years investigated by overnight polysomnography (PSG) or overnight respiratory recording for suspected OSAHS (snoring, apnea reported by the bed partner, some forms of daytime sleepiness) in 7 sleep centers from the west of France are eligible for inclusion in a prospective cohort, the IRSR sleep cohort [21, 22]. Patients with mental retardation, patients unable to fill in the questionnaires, patients unable to read and/or speak French, and patients with neuromuscular diseases are excluded from the IRSR sleep cohort. In this cohort, between May 15, 2007 and December 1^st^, 2014, 4,547 patients were diagnosed with OSAHS. Only 3 of the 7 centers propose MAD as an alternative to CPAP. All consecutive patients in these 3 centers (n = 2,822), in whom CPAP or MAD was prescribed were eligible for inclusion in this study.

### Baseline evaluation

Baseline evaluation prior to treatment initiation included recording of patient characteristics, associated cardiovascular morbidities and OSAHS disease severity. Patients filled in questionnaires evaluating subjective daytime sleepiness, depressive symptoms and socioeconomic factors.

#### Patient characteristics

Patients were characterized according to their age (<55/≥55 years), gender, body mass index (BMI) (<30/≥30 kg/m^2^), smoking (non-current smoker/current smoker), and alcohol consumption habits (< 3 glasses of wine per day /≥ 3 glasses of wine per day).

#### Associated cardiovascular morbidities—hypertension—diabetes

Patients were classified as having cardiovascular morbidity if they reported at least one of the following cardiovascular diseases: ischemic heart disease, cardiac arrhythmia, congestive heart failure or stroke.

Patients were classified as having hypertension if they reported treatment for hypertension.

Patients were classified as having diabetes if they reported treatment for type I or II diabetes.

#### OSAHS disease severity

Subjects were stratified by OSAHS severity based on an AHI cutoff of 30 (AHI <30/≥30 events per hour) measured by overnight PSG or overnight respiratory recording. Respiratory events were scored manually. The criterion used to define an apnea event is a cessation of airflow lasting 10 seconds or longer; a 50% reduction of airflow or a discernable reduction of airflow associated with micro-arousals and /or a 3% or more reduction in oxyhemoglobin saturation defined a hypopnea event.

#### Subjective daytime sleepiness

Excessive daytime sleepiness was defined by an Epworth Sleepiness Scale (ESS) ≥10 [23].

#### Depressive symptoms

Depression was diagnosed when at least 7 items of the 13-item version of the Pichot depression scale [24] were positive.

#### Socioeconomic factors

Using specifically designed self-administered questionnaires from the Institut National de la Statistique et des Etudes Economiques (INSEE), socioeconomic status was described by the following variables: marital status (married or living as a couple/living alone [never married, divorced, separated, widowed]); employment status (employed full time or part time/ retired/unemployed); educational attainment as determined by the age at which the patient left full-time education (<18/≥18 years); the patient’s last occupation according to the INSEE nomenclature (farmers/craftsmen/shopkeepers, executives and higher intellectual professions /Intermediate professions, technicians, foremen/employees/workers) [25]; and private health insurance (yes/no).

### Primary dependent variable

The primary dependent variable was ‘being treated by MAD’ or not. If patients were not treated by MAD they were treated by CPAP.

### Independent variables of primary interest and confounding variables

The independent variables of primary interest were socioeconomic factors. The other variables, including patient characteristics, associated cardiovascular morbidities, OSAHS disease severity, subjective daytime sleepiness, depressive symptoms, constituted confounding variables.

### Statistical analysis

Patients treated by MAD and those treated by CPAP were compared using Chi-square test for categorical variables and 2-sample t-test for continuous variables. The interaction between the educational attainment and patient’s last occupation was tested. Logistic regression and marginal logistic regression with center as cluster variable were then performed, using a stepwise forward procedure with a p value of 0.05. Results were expressed as adjusted odds ratios (aOR) (95% confidence intervals). All statistical analyses were performed with SAS software (SAS/ STAT Package 2002–2003 by SAS Institute Inc., Cary, NC, USA).

## Results

Of the 2,822 patients included in the study, 351 (12.4%) were treated by MAD and 2,471 (87.6%) were treated by CPAP.

### Univariate analysis

Comparison of patients treated by MAD with those treated by CPAP demonstrated significant differences. Patients treated by MAD were younger, had a lower BMI, less associated co-morbidities (cardio-vascular morbidities, diabetes, hypertension), and less severe depression score. OSAH was less severe in this group, with an AHI more frequently <30. They were less retired or unemployed than those treated with CPAP. They were also higher educated and were over represented among executives and higher intellectual professions (Table 1).

**Table 1:** Baseline characteristics of patients treated by MAD and patients treated by CPAP. Results are presented as percentages unless otherwise indicated. Significant level for p value: <0.05.

	Patients treated by MAD (n = 351)	Patients treated by CPAP (n = 2471)	P value
**Female**	29.6	30.2	0.8184
**Age, mean (SD)**	55.0 (11.5)	59.8 (12.9)	<0.0001
**Age ≥ 55 years**	51.3	65.1	<0.0001
**Married or living as a couple**	82.8	78.6	0.0747
**Body mass index, mean (SD)**	26.3 (3.6)	32.9 (6.9)	<0.0001
**Body mass index ≥ 30 kg/m** ^**2**^	13.4	63.2	
**Left full time education ≥ 18**	74.2	29.1	<0.0001
**Employment status**			<0.0001
Employed full time or part time	68.9	45.1	
Retired	22.6	40.2	
Unemployed	8.5	14.7	
Patients who left full-time education ≥ 18 years	64.2	29.1	<0.0001
**Last occupation**			<0.0001
Farmers/Craftsmen/shopkeepers	9.5	14.0	
Executives and higher intellectual professions	38.8	17.2	
Intermediate professions, technicians, foremen	25.4	21.7	
Employees	14.4	17.8	
Workers	11.9	29.4	
**Private health insurance: yes**	98.8	98.2	0.1011
**Epworth sleepiness score ≥ 10**	48.6	53.9	0.06
**Apnea-hypopnea index ≥ 30**	46.8	84.1	<0.0001
**Pichot depression score ≥ 7**	13.2	23.3	<0.0001
**Patients with cardiovascular morbidity**	4.6	16.9	<0.0001
**Patients treated for type I or II diabetes**	3.3	21.0	<0.0001
**Patients treated for hypertension**	21.0	43.0	<0.0001
**Alcohol ≥ 3 glasses of wine per day**	50.0	46.2	0.1910
**Current smokers**	31.6	35.7	0.2413

### Multivariate analysis

All results of the stepwise regression analysis are summarized in Table 2. The main outcome of the study was that treatment by MAD was significantly associated with both educational attainment, as determined by the age at which the patient left full-time education, ≥18 years compared with <18 (aOR: 1.64, 95% CI 1.23 to 2.20), and the patient’s occupational category. Executives and higher intellectual professions, intermediate professions, technicians, foremen and employees were significantly more likely to be treated by MAD than workers (aOR: 2.21, 95% CI 1.88 to 2.58; aOR: 1.74, 95% CI 1.15 to 2.63; aOR: 1.96, 95% CI 1.11 to 3.47, respectively). The interaction between the educational attainment and patient’s last occupation was not statistically significant (p = 0.9). All results of the stepwise marginal regression analysis with center as a cluster variable are summarized in Table 3. The estimates were very similar between the two models, eliminating a center effect.

**Table 2:** Stepwise regression analysis of variables influencing MAD therapy (n = 2822).

	Coefficient (standard error)	Odds Ratio (confidence interval)
**Age (ref: ≥ 55 years)**		
< 55 years	0.3581 (0.1024)	1.43 (1.17–1.75)
**Body Mass Index (BMI) (ref: BMI≥30)**		
BMI<30	1.5888 (0.2600)	4.90 (2.94–8.15)
**Educational attainment (ref: left full-time education < 18 years)**		
Left full-time education ≥ 18 years	0.4958 (0.1490)	1.64 (1.23–2.20)
**Last occupation (ref: workers)**		
Farmers/Craftsman/shopkeepers	0.4047 (0.2245)	1.50 (0.97–2.33)
Executives and higher intellectual professions	0.7911 (0.0803)	2.21 (1.88–2.58)
Intermediate professions, technicians, foremen	0.5523 (0.2111)	1.74 (1.15–2.63)
Employees	0.6740 (0.2907)	1.96 (1.11–3.47)
**Sleepiness (ref: Epworth sleepiness score ≥ 10)**		
Epworth sleepiness score <10	0.3819 (0.1668)	1.47 (1.06–2.03)
**Apnea-hypopnea index (AHI) (ref: AHI ≥ 30)**		
AHI <30	1.2891 (0.1779)	3.63 (2.56–5.14)
**Depression (ref: Pichot depression score ≥ 7)**		
Pichot depression score <7)	0.6372 (0.0587)	1.89 (1.69–2.12)
**Patients with cardiovascular morbidity (ref: yes)**		
No	1.0185 (01836)	2.77 (1.93–3.97)
**Patients with treated diabetes I or II (ref: yes)**		
No	1.0694 (0.4807)	2.91 (1.14–7.48)

**Table 3:** Stepwise marginal regression analysis of variables influencing MAD therapy with center as a cluster variable (n = 2822).

	Coefficient (standard error)	Odds Ratio (confidence interval)
**Age (ref: ≥ 55 years)**		
< 55 years	0.3701 (0.1206)	1.45 (1.14–1.83)
**Body Mass Index (BMI) (ref: BMI≥30)**		
BMI<30	1.6688 (0.3133)	5.30 (2.87–9.80)
**Educational attainment (ref: left full-time education < 18 years)**		
Left full-time education ≥ 18 years	0.5203 (0.1790)	1.68 (1.18–2.39)
**Last occupation (ref: workers)**		
Farmers/Craftsman/shopkeepers	0.4076 (0.2588)	1.50 (0.91–2.49)
Executives and higher intellectual professions	0.8222 (0.0896)	2.28 (1.91–2.71)
Intermediate professions, technicians, foremen	0.5772 (0.2304)	1.78 (1.13–2.80)
Employees	0.7078 (0.3212)	2.03 (1.08–3.81)
**Sleepiness (ref: Epworth sleepiness score ≥ 10)**		
Epworth sleepiness score <10	0.4021 (0.1879))	1.50 (1.03–2.16)
**Apnea-hypopnea index (AHI) (ref: AHI ≥ 30)**		
AHI <30	1.3048 (0.1692)	3.69 (2.65–5.14)
**Depression (ref: Pichot depression score ≥ 7)**		
Pichot depression score <7)	0.5429 (0.1023)	1.72 (1.41–2.10)
**Patients with cardiovascular morbidity (ref: yes)**		
No	1.0562 (0.2112)	2.88 (1.90–4.35)
**Patients with treated diabetes I or II (ref: yes)**		
No	1.0922 (0.5463)	2.98 (1.02–8.70)

## Discussion

Overall, the determinants of being treated with MAD are related to both disease severity and the patient’s socioeconomic status. All things being equal, a diagnosis of mild or moderate OSAHS (i.e. AHI<30 and Epworth Score<10 with no cardiovascular comorbidity) was associated with MAD therapy. These results are not surprising and tend to confirm that these prescribing practices comply with French and international guidelines [26]. CPAP should be proposed as first-line treatment in patients with severe OSAHS (AHI>30), and MAD should only be proposed to patients who refuse do not tolerate CPAP. It must be noted that disease severity criteria were included as potential confounders in the model. More interestingly, this study demonstrates for the first time that, after adjusting for disease severity, the patient’s socioeconomic characteristics such as their educational attainment and their last occupational category had a major impact on the choice of treatment. If the patient left full-time education ≥18 years and was not a “worker”, he or she was more likely to be treated with MAD.

This last result confirms our baseline hypothesis that the patients’ socioeconomic conditions influence how they are treated. MAD and CPAP devices are both 60% reimbursed by French national health insurance (public insurance). The remaining 40%, representing €136 and €374 per year, respectively, must be paid by the patient or by private health insurance depending on the level of coverage. At first glance, these figures might appear to be in favor of MAD, but such an interpretation fails to see the forest for the trees: Repeated visits to the dentist are necessary to fit the MAD and ensure optimal adaptation of the appliance, which can take several weeks and cost more than 300 euros (out-of-pocket by the patient). MAD therapy also requires a minimum number of healthy teeth and good oral hygiene. As already mentioned, oral health and access to dental care remain two important issues in France. In a study that systematically examined the rate of primary CIs to MAD in a group of 100 consecutive patients with OSAHS referred to a tertiary sleep center in France, CIs to MAD were present in 34% of patients, mainly related to an insufficient number of teeth and periodontal disease combined with tooth mobility. Another subgroup of patients (15%) required very close follow-up during the first weeks of treatment to prevent exacerbation of preexisting dental problems [15]. Most of these initial CIs of MAD could be removed if the proper dental care was performed. The dental care required to restore good dental conditions is very expensive. In France, not all forms of dental care are publicly subsidized. Teeth scaling, caries treatment, and dental extractions are reimbursed at 70% by the French national health insurance on the basis of a fixed rate that the dentist cannot exceed. The remaining 30% of the cost is covered by supplementary insurance and/or out-of-pocket by the patient. In contrast, the cost to be charged by the dentist is not restricted for dental prostheses, i.e. dental crowns, bridges, and implants. It is precisely these treatments that patients may need to establish optimal oral health that carry a high cost burden. One can hypothesize that sleep physician incorporates this economical constraint in his/her therapeutic decision by choosing the CPAP in patients with low SES.

Oral health is subject to marked social inequalities that can be explained by behavioral factors (hygiene, food, etc.) as well as financial barriers [16,18]. Behavioral factors, such as cultural and information barriers, can explain why the poorest and least educated individuals less frequently seek medical care, and tend to seek curative rather than preventive care. These differences would be due to a poorer knowledge about healthcare pathways, a different relationship with the body or the disease and different priorities concerning the degree of time preference, risk and importance attributed to health [19]. Financial barriers are also very important. Individuals who do not seek treatment are mostly those with fewer resources or without private health insurance. Based on the data of the “Enquête santé protection sociale” survey, 15.4% of the adult population reported that they had already renounced care for financial reasons, particularly for dental care (10% of the population) [27]. In this study, health care renunciation was almost twice as frequent in the population without private health insurance or with poor insurance cover compared to the population with good or fairly good private health insurance coverage [28]. Patient income is not routinely recorded in the IRSR sleep cohort, but educational level and occupational category can be considered to constitute good surrogate markers [29]. In the present study, 98% of patients were covered by private health insurance, with no significant difference between those treated by MAD and those treated by CPAP, but the effect of private health insurance on treatment choice could not be tested due to the absence of data concerning reimbursement rates.

Ultimately, as MAD is better tolerated than CPAP, where patients have higher compliance rates, it translates into a similar mean disease alleviation (defined as a combined function of efficacy and compliance) [30]. Thus, patients with low SES having poor dental conditions are doubly penalized: 1) they cannot benefit from MAD treatment, and 2) without any choice other than CPAP, they are potentially at risk of being less adherent to treatment [21].

This study presents a number of limitations. First of all, no information was available concerning the patients’ oral hygiene, preventing analysis of the confounding role of oral hygiene on the relationship between socioeconomic factors and choice of treatment. However, we did not find any other convincing reasons to explain our findings. Poor dental condition represents actually a contraindication to MAD therapy. If we had been able to adjust for dental conditions, the effect of the patients’ socioeconomic level may have persisted as a result of cultural or information barriers, as mentioned at the beginning of the discussion section, making these patients less aware of the alternative innovative treatments for OSAHS such as MAD. The second limitation is that only patient variables were included in the model. The attitude of physicians in relation to a treatment not considered to be the "gold standard" should also be taken into account. Physician-related variables were not available in the IRSR sleep cohort. Centre-related variability was considered by using a marginal logistic regression model with center as cluster variable.

## Conclusions

In conclusion, this study shows, for the first time, that socioeconomic factors are determinants in the choice of MAD for OSAHS. Because MAD therapy is effective and simple to use, it may avoid the disadvantages of CPAP under certain conditions. However, MAD can only be prescribed to patients with satisfactory oral hygiene, which unfortunately remains a major health issue in France and in other OECD countries. Only the most privileged part of the population has access to oral care. MAD therapy used to treat OSAHS actually highlights inequalities in health care use.
